# Supplementary material for: Molecular and metabolomic characterization of hiPSC-derived cardiac fibroblasts transitioning to myofibroblasts
Source: Front Cell Dev Biol. 2024 Dec 4;12:1496884. doi: 10.3389/fcell.2024.1496884 (PMC11653212; doi:10.3389/fcell.2024.1496884)
Supplement: Supplementary file 1 [file Table1.docx]

**Supplemental Table 1.** Primers used for quantitative real-time PCR. Forward (F) and reverse (R) primers are shown.

| ED-A-Fn-F (human) | 5’- ACTGCAGTAACCAACATTGATC -3’ |
| --- | --- |
| ED-A-Fn-R (human) | 5’- CACCCTGTACCTGGAAACTTGC -3’ |
| Postn-F (human) | 5’- TGTTGCCCTGGTTATATGAG -3’ |
| Postn-R (human) | 5’- GTGGTGGCTCCCACGATGCC -3’ |
| TCF21-F (human) | 5’- CACTTGAGGCAGATCCTGGCTA -3’ |
| TCF21-R (mouse) | 5’- CGGTCACCACTTCTTTCAGGTC -3’ |
| β-actin-F (human) | 5’- ATTGCCGACAGGATGCAGAA -3’ |
| β-actin-R (human) | 5’- GGGCCGGACTCGTCATACTC -3’ |
