# Supplementary material for: Molecular and metabolomic characterization of hiPSC-derived cardiac fibroblasts transitioning to myofibroblasts
Source: Front Cell Dev Biol. 2024 Dec 4;12:1496884. doi: 10.3389/fcell.2024.1496884 (PMC11653212; doi:10.3389/fcell.2024.1496884)
Supplement: Supplementary file 2 [file DataSheet1.pdf]

Supplemental Figure Captions

Figure S-1: Passaging influences markers of fibroblast proliferation. Violin plots represent mRNA expression of CDK4 (A), and proteomic expression of PCNA (B) MCM3 (C) and CDK1 (D), showed reduction in the proliferation in P3 and P0+TGFβ1. Statistical significance was determined by one-way ANOVA with Tukeys post-hoc test (n=4-5) *$p \leq 0.05$, **$p \leq 0.01$, ***$p \leq 0.001$, ****$p \leq 0.0001$, vs fibroblasts (P0) and ##$p \leq 0.01$, vs P0+TGFβ1. iPS IMR90-1 and STAN248i-617C1 lines were used to generate the data for **Fig. S-1-A.** iPS IMR90-1 line was used to generate the data for **Fig. S-1-B-D.**

Figure S-2: Passaging augments ECM remodeling enzyme expression. Violin plots represent mRNA expression of LOX (A), MMP-2 (B) MMP-1(C) and proteomic expression of LOX (D), and PLOD2 (E), showing elevated ECM remodeling enzymes as an indirect measure of collagen synthesis in P3 and P0+TGFβ1 compared to P0. Statistical significance was determined by one-way ANOVA with Tukey's post-hoc test (n=4-5) *$p \leq 0.05$, **$p \leq 0.01$, ***$p \leq 0.001$, ****$p \leq 0.0001$, vs fibroblasts (P0) and ####$p \leq 0.0001$ vs P0+TGFβ1. iPS IMR90-1 and STAN248i-617C1 lines were used to generate the data for **Fig. S-2-A-C.** iPS IMR90-1 line was used to generate the data for **Fig. S-2-D-E.**

Figure S-3: Passaging promotes profibrotic gene expression in myofibroblasts. Violin plots of profibrotic gene expression using Nanostring analysis in P0+TGFβ1 and P3 compared to P0, (A-I); Statistical significance was determined by one-way ANOVA with Tukey's post-hoc test (n=4-5). *$p<0.05$, **$p<0.01$, ***$p<0.001$, ****$p<0.0001$ vs fibroblasts (P0) and #$p \leq 0.05$, ##$p \leq 0.01$, ####$p \leq 0.0001$ vs P0+TGFβ1. iPS IMR90-1 and STAN248i-617C1 lines were used to generate the data for **Fig. S-3-A-I.**

Figure S-4: Passage-induced fibroblast activation and mitochondrial metabolism. Seahorse assay reveals that coupling efficiency (A) showed no differences regardless of passage or treatment also, non-mitochondrial OCR (B) varied with passage (P3) and/or TGFβ1 treatment, whereas the glutaminase inhibitor BPTES inhibited this process when compared to non-passaged fibroblasts (P0). Statistical significance was determined by one-way ANOVA with Tukey's post-hoc test (n = 3-8); *$p \leq 0.05$, **$p \leq 0.01$, ***$p \leq 0.001$, ****$p \leq 0.0001$, vs fibroblasts (P0) and #$p \leq 0.05$, ####$p \leq 0.0001$ vs P0+TGFβ1. iPS IMR90-1 and STAN248i-617C1 lines were used to generate the data for **Fig. S-4-A, B.**

Figure S1

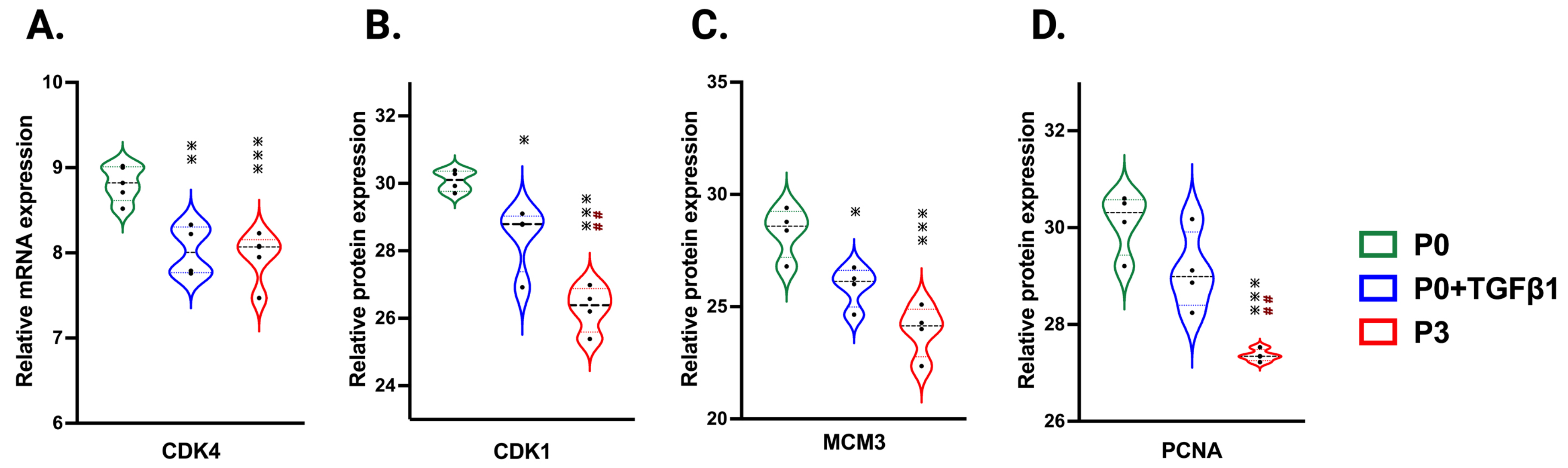

A.
B.
C.
D.
Relative mRNA expression
10
9
8
7
6
CDK4
Relative protein expression
32
30
28
26
24
CDK1
35
30
25
20
MCM3
32
30
28
26
PCNA
P0
P0+TGFβ1
P3

Figure S2.

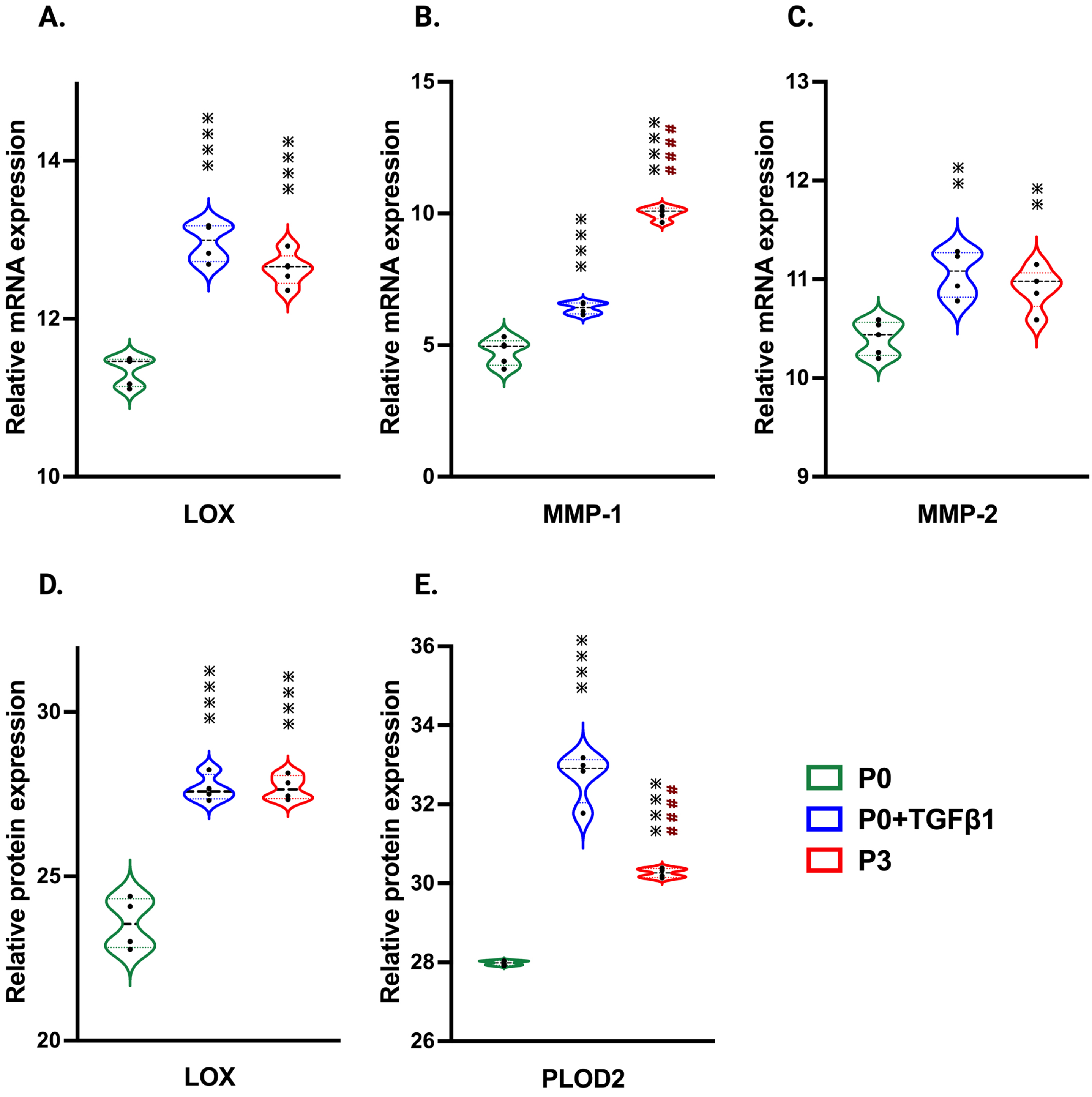
A.
B.
C.
D.
E.
Relative mRNA expression
Relative protein expression
LOX
MMP-1
MMP-2
PLOD2
P0
P0+TGFβ1
P3
****
**
####

Figure S3.

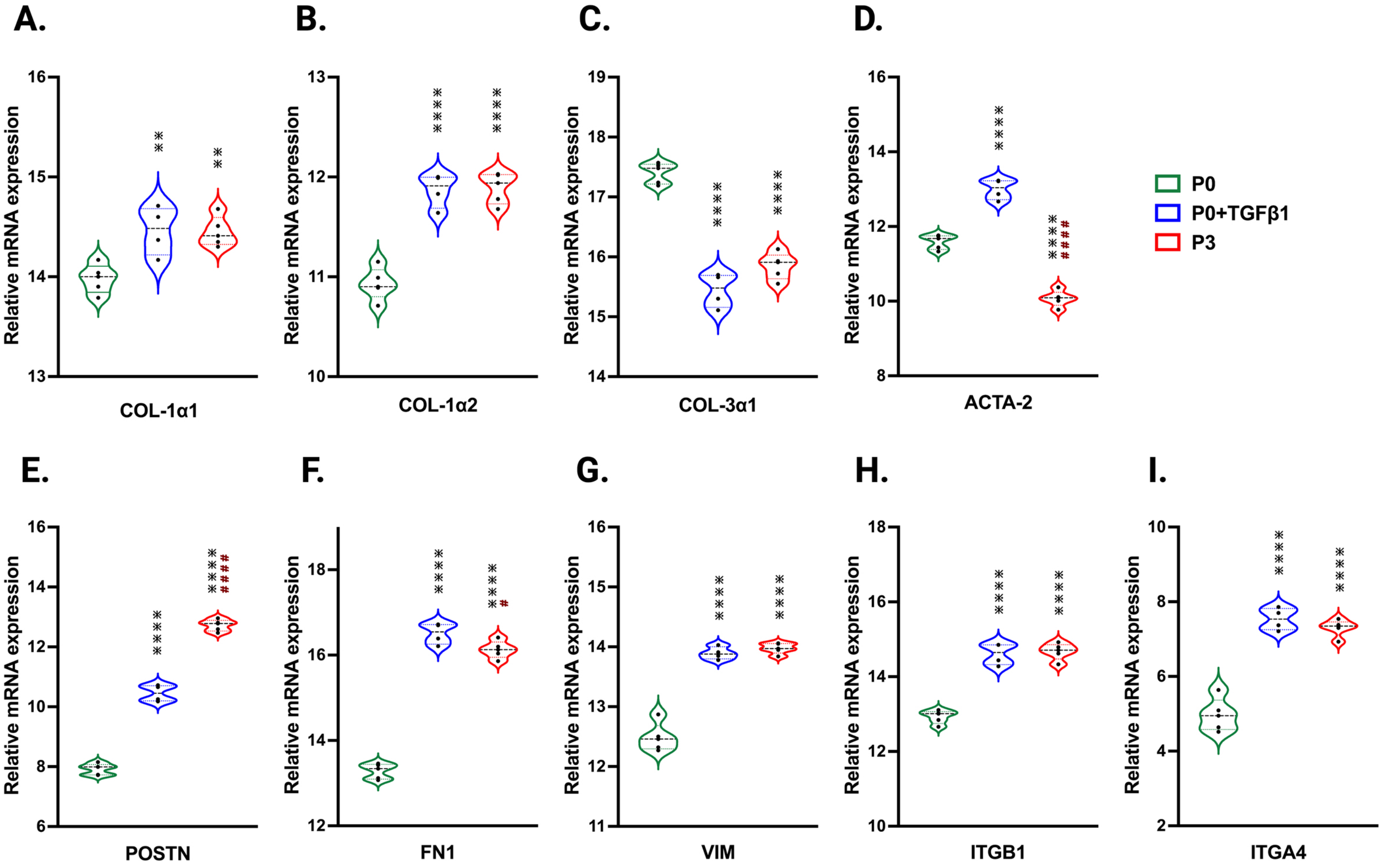
A.
B.
C.
D.
E.
F.
G.
H.
I.
Relative mRNA expression
COL-1α1
COL-1α2
COL-3α1
ACTA-2
POSTN
FN1
VIM
ITGB1
ITGA4
P0
P0+TGFβ1
P3

Figure S4.

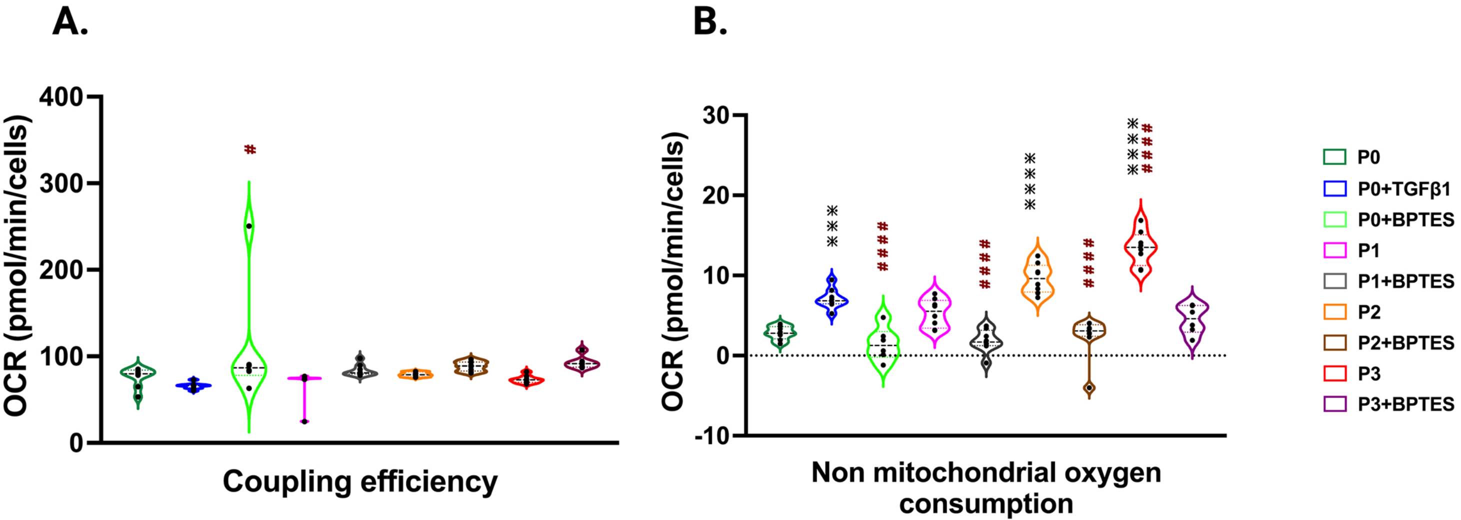

A.
OCR (pmol/min/cells)
400
300
200
100
0
#
Coupling efficiency
B.
OCR (pmol/min/cells)
30
20
10
0
-10
Non mitochondrial oxygen consumption
P0
P0+TGFβ1
P0+BPTES
P1
P1+BPTES
P2
P2+BPTES
P3
P3+BPTES
